# New records of megophryids (Amphibia: Anura: Megophryidae) from Son La Province, Vietnam

**DOI:** 10.3897/BDJ.7.e39140

**Published:** 2019-11-07

**Authors:** Anh Van Pham, Cuong The Pham, Lan Duc Doan, Thomas Ziegler, Truong Quang Nguyen

**Affiliations:** 1 Faculty of Biology and Chemistry, Tay Bac University, Son La City, Son La Province, Vietnam Faculty of Biology and Chemistry, Tay Bac University Son La City, Son La Province Vietnam; 2 Institute of Ecology and Biological Resources, Vietnam Academy of Science and Technology, Hanoi, Vietnam Institute of Ecology and Biological Resources, Vietnam Academy of Science and Technology Hanoi Vietnam; 3 Tay Bac University, Son La City, Son La Province, Vietnam Tay Bac University Son La City, Son La Province Vietnam; 4 Cologne Zoo, Cologne, Germany Cologne Zoo Cologne Germany; 5 Institute of Zoology, University of Cologne, Cologne, Germany Institute of Zoology, University of Cologne Cologne Germany; 6 Graduate University of Science and Technology, Vietnam Academy of Science and Technology, Hanoi, Vietnam Graduate University of Science and Technology, Vietnam Academy of Science and Technology Hanoi Vietnam

**Keywords:** Distribution, morphology, taxonomy.

## Abstract

**Background:**

The family Megophryidae is one of the most poorly known groups of amphibians in Son La Province, Vietnam. In the herpetofaunal list of Vietnam in 2009 only two species of megophryids were recorded from this province and recent studies have documented five additional species of Megophryidae from Son La Province.

**New information:**

Based on recent field work in northern Vietnam, we report six new provincial records of the family Megophryidae from Son La Province, namely *Leptobrachium ailaonicum*, *Leptobrachella sungi*, *Megophrys feae*, *M. jingdongensis*, *M. microstoma* and *M. parva*. In addition, morphological data were provided for each species, based on newly collected specimens. Our findings bring the species number of the family Megophryidae to 13 in Son La Province.

## Introduction

The family Megophryidae is one of the most poorly studied groups of amphibians in Vietnam. The knowledge about species diversity of this family in Vietnam has remarkably increased from 25 in 2009 to 57 at present (Nguyen et al. 2009, Frost 2019). Sixteen new species have been recently described in the last five years, namely *Leptobrachella ardens* (Rowley, Tran, Le, Dau, Peloso, Nguyen, Hoang, Nguyen & Ziegler), *Leptobrachella isos* (Rowley, Stuart, Neang, Hoang, Dau, Nguyen & Emmett), *Leptobrachella kalonensis* (Rowley, Tran, Le, Dau, Peloso, Nguyen, Hoang, Nguyen & Ziegler), *Leptobrachella macrops* Duong, Do, Ngo, Nguyen & Poyarkov, *Leptobrachella maculosa* (Rowley, Tran, Le, Dau, Peloso, Nguyen, Hoang, Nguyen & Ziegler), *Leptobrachella pallida* (Rowley, Tran, Le, Dau, Peloso, Nguyen, Hoang, Nguyen & Ziegler), *Leptobrachella petrops* (Rowley, Dau, Hoang, Le, Cutajar & Nguyen), *Leptobrachella puhoatensis* (Rowley, Dau & Cao), *Leptobrachella pyrrhops* (Poyarkov, Rowley, Gogoleva, Vassilieva, Galoyan & Orlov), *Leptobrachella rowleyae* (Nguyen, Poyarkov, Le, Vo, Ninh, Duong, Murphy & Nguyen), *Leptobrachella tadungensis* (Rowley, Tran, Le, Dau, Peloso, Nguyen, Hoang, Nguyen & Ziegler), *Megophrys elfina* Poyarkov, Duong, Orlov, Gogoleva, Vassilieva, Nguyen, Nguyen, Nguyen, Che & Mahony, *Megophrys fansipanensis* Tapley, Cutajar, Mahony, Nguyen, Dau, Luong, Le, Nguyen, Nguyen, Portway, Luong & Rowley, *Megophrys hoanglienensis* Tapley, Cutajar, Mahony, Nguyen, Dau, Luong, Le, Nguyen, Nguyen, Portway, Luong & Rowley, *Megophrys latidactyla* Orlov, Poyarkov & Nguyen, and *Megophrys rubrimera* Tapley, Cutajar, Mahony, Chung, Dau, Nguyen, Luong & Rowley (Poyarkov et al. 2017, Frost 2019). In addition, two species were recently recorded for the first time from Vietnam: *Megophrys daweimontis* Rao & Yang and *Megophrys synoria* (Stuart, Sok & Neang) (Le et al. 2014, Vassilieva et al. 2016).

In Son La Province, Nguyen et al. (2009) recorded only two species of the family Megophryidae, namely *Leptobrachium chapaense* (Bourret) and *Megophrys major* Boulenger; Pham et al. (2014b) recorded three additional species, namely *M. pachyproctus* Huang, *M. palpebralespinosa* Bourret, and *Leptobrachella ventripunctata* (Fei, Ye & Li). Pham et al. (2016) reported two species for the first time from this province, viz. *Leptobrachium masatakasatoi* Matsui and *Leptobrachella minima* (Taylor). Based on our recent field work in Son La Province between 2014 and 2017, we herein report six new provincial records of the family Megophryidae.

## Materials and methods

### Sampling

**Figure 1.**
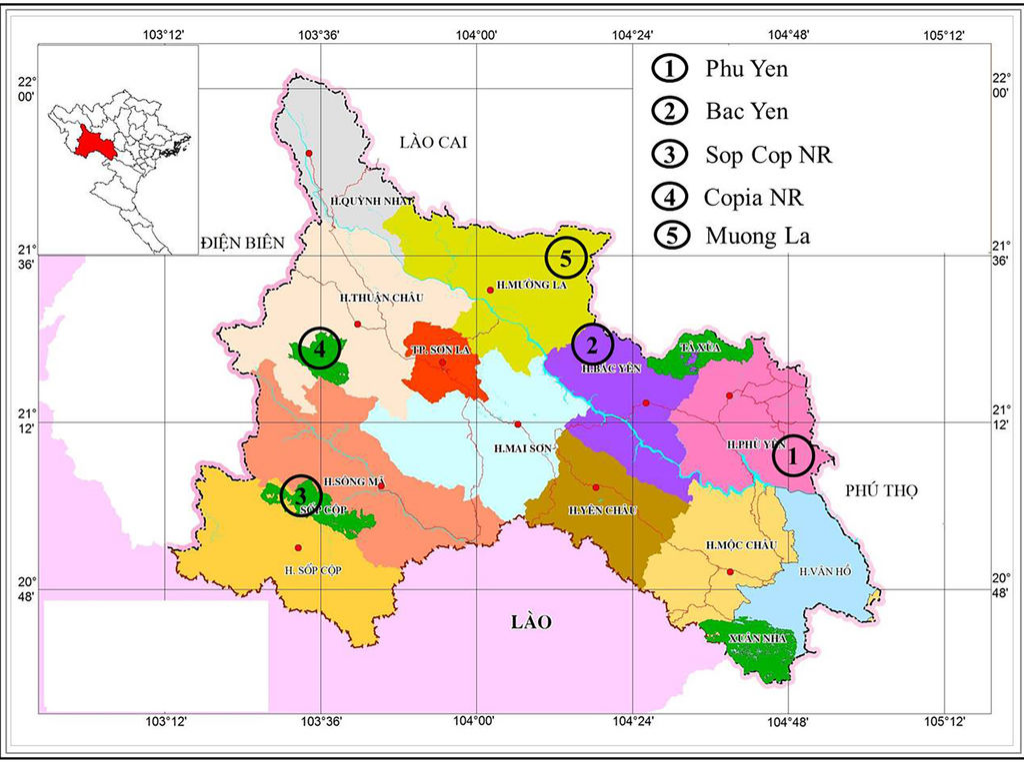
Map showing the sampling sites in Son La Province, northwestern Vietnam

Field surveys were conducted in Son La Province from 18 to 22 June 2014 in Copia Nature Reserve (NR); from 27 to 29 April 2015 in Sop Cop NR; from 28 August to 4 September 2015 and from 26 to 30 October 2016 in Phu Yen District; from 14 to 17 April 2017 in Muong La NR; and from 28 April to 3 May 2017 in Bac Yen District (Fig. 1).

Specimens were collected by hand between 19:00–22:00 h. After taking photographs, specimens were euthanised in a closed vessel with a piece of cotton wool containing ethyl acetate (Simmons 2002), fixed in 80% ethanol for five hours and then transferred to 70% ethanol for permanent storage. Voucher specimens were subsequently deposited in the collections of the Institute of Ecology and Biological Resources (IEBR), Hanoi and the Tay Bac University (TBU), Son La Province, Vietnam.

### Morphological characters

Measurements were taken with a digital caliper to the nearest 0.1 mm. Abbreviations are as follows: SVL: snout–vent length; HL: head length from posterior corner of mandible to tip of snout; HW: maximum head width, at the angle of jaws; IN: internarial distance; SL: distance from anterior corner of eye to tip of snout; NS: distance from anterior edge of nostril to tip of snout; EN: distance from anterior corner of eye to posterior edge of nostril; EL: eye length, from anterior corner to posterior corner of eye; IOD: minimum distance between upper eyelids; UEW: maximum width of upper eyelid; TD: maximum tympanum diameter; FLL: forelimb length, from axilla to tip of third finger; HLL: hind limb length, from vent to tip of fourth toe; FL: thigh length, from vent to knee; TL shank length. For webbing formula, we followed Glaw and Vences (2007). Sex was determined by gonadal inspection.

## Taxon treatments

### Leptobrachium ailaonicum

(Yang, Chen & Ma, 1983)

2A19B8F9-DB7B-58DD-94E3-461390C40B6A

#### Materials

- **Type status:** Other material. **Occurrence:** catalogNumber: TBU ML.2016.30; individualCount: 1; sex: male; lifeStage: adult; **Taxon:** scientificNameID: Leptobrachium ailaonicum; scientificName: Leptobrachium ailaonicum; class: Amphibia; order: Anura; family: Megophryidae; genus: Leptobrachium; specificEpithet: ailaonicum; scientificNameAuthorship: (Yang, Chen & Ma, 1983); **Location:** country: Vietnam; countryCode: VN; stateProvince: Son La; county: Muong La; municipality: Ngoc Chien; locality: Muong La Nature Reserve, near Nam Nghep Village; verbatimElevation: 2010 m; verbatimLatitude: 21°19.66'N; verbatimLongitude: 103°36.26'E; verbatimCoordinateSystem: WGS84; **Event:** eventDate: October 7, 2016; eventRemarks: collected by N.B. Sung and T.V. Dau; **Record Level:** language: en; collectionCode: Amphibians; basisOfRecord: Preserved Specimen
- **Type status:** Other material. **Occurrence:** catalogNumber: TBU SL.2016.513; individualCount: 1; sex: male; lifeStage: adult; **Taxon:** scientificNameID: Leptobrachium ailaonicum; scientificName: Leptobrachium ailaonicum; class: Amphibia; order: Anura; family: Megophryidae; genus: Leptobrachium; specificEpithet: ailaonicum; scientificNameAuthorship: (Yang, Chen & Ma, 1983); **Location:** country: Vietnam; countryCode: VN; stateProvince: Son La; county: Muong La; municipality: Ngoc Chien; locality: Muong La Nature Reserve, near Nam Nghep Village; verbatimElevation: 2011 m; verbatimLatitude: 21°19.66'N; verbatimLongitude: 103°36.26'E; verbatimCoordinateSystem: WGS84; **Event:** eventDate: October 28, 2016; eventRemarks: collected by N.B. Sung and T.V. Dau; **Record Level:** language: en; collectionCode: Amphibians; basisOfRecord: Preserved Specimen

#### Description

Morphological characters of specimens (n = 2 males) from Son La Province agreed with the descriptions of Yang et al. (1983) and Ho et al. (1999): SVL 71.4–75.8 mm; head wider than long (HL 26.1–28.1 mm, HW 28.5–31.8 mm, HL/SVL 37%; HW/SVL 40.0–42.0%); snout round (SL 11.0–11.3 mm), longer than horizontal diameter of eye (EL 9.5–9.9 mm); canthus rostralis distinct; loreal region oblique, moderately concave; nostril lateral, closer to tip of snout than to eye (NS 4.8–4.9 mm, EN 5.2–5.5 mm); interorbital space flat, broader than upper eyelid and internarial distance (IOD 9.5–9.8 mm, UEW 7.3–7.4 mm, IN 5.0–5.2 mm); tympanum indistinct; vomerine teeth absent; tongue heart-shaped, notched posteriorly; vocal openings absent.

Forelimb long (FLL 58.0–62.8 mm); relative finger lengths II < I < IV < III; fingers without dermal fringe, free of webbing; tips of fingers rounded, slightly swollen; subarticular tubercle indistinct; palmar tubercles two, oval; nuptial pads absent.

Hindlimb slender, long (HLL 105.0–106.7 mm); thigh longer than tibia (FL 34.8–36.6, TL 31.3–34.0 mm); tips of toes slightly swollen; webbing formula I1–2II1–21/2III2–3/1/2IV4–2V; inner metatarsal tubercle distinct, shorter than length of toe I; tibiotarsal articulation reaching to posterior margin of orbit when limb adpressed along body.

Skin. Dorsal surface with fine network of ridges, tubercles present in the posterior region of tympanum; upper lip with 46–78 keratinised spines; supratympanic fold present, from posterior edge of eye to axilla; flanks, belly, chest, throat, underside of forelimbs and thighs with small white pustules.

**Figure 2.**
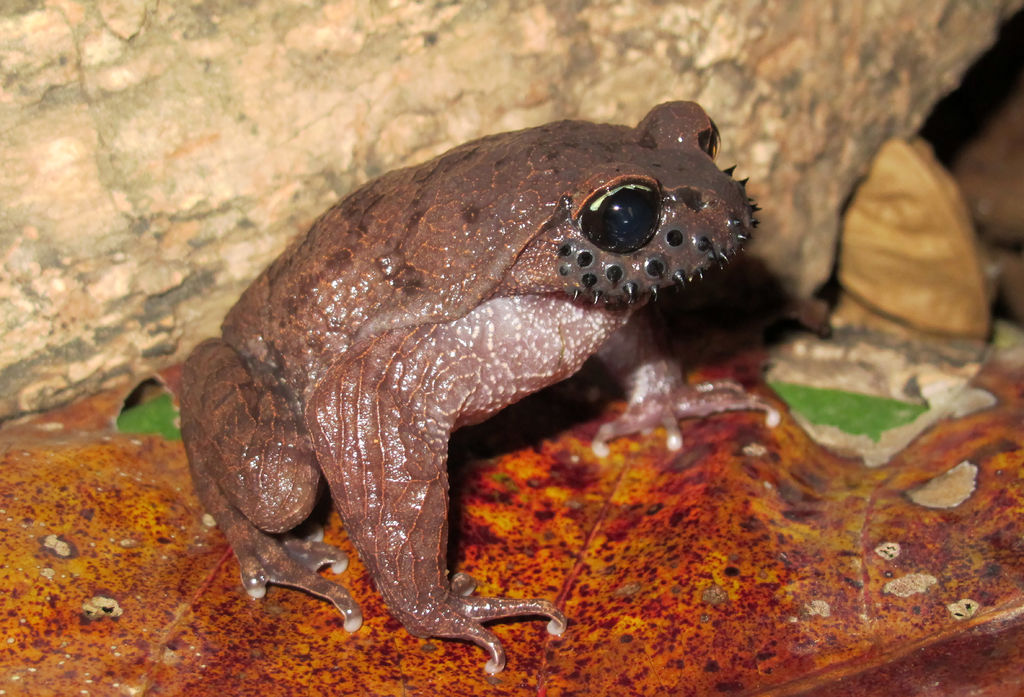
*Leptobrachium ailaonicum* (TBU ML.2017.9, adult male) from Son La Province, Vietnam. Photo by A.V. Pham.

Colouration in life. Dorsal surface of the head light brown or reddish-brown, with dark brown spots on medial side of upper eyelid; back with irregularly dark brown spots; ventral surface with light brown pustules, more concentrated in chest area (Fig. 2).

#### Distribution

In Vietnam, this species was recorded from Lao Cai Province (Nguyen et al. 2009). Our new record is approximately 70 km distant from Lao Cai Province. Elsewhere, this species is known from China (Frost 2019).

#### Ecology

The specimens of *L. ailaonicum* were found on the edge of small streams and on forest paths between 20:00 and 21:00 h. The surrounding habitat was mixed secondary forest of large hardwood and shrub.

#### Taxon discussion

The male specimens from Son La have more keratinied spines than those in the previous studies: 46–78 versus 20–48 (Yang et al. 1983) and 50–59 (Ho et al. 1999).

### Leptobrachella sungi

(Lathrop, Murphy, Orlov & Ho, 1998)

038B5AFB-55CD-5F6E-9B38-018FF182D559

#### Materials

- **Type status:** Other material. **Occurrence:** catalogNumber: TBU MD.2016.116; individualCount: 1; sex: female; lifeStage: adult; **Taxon:** scientificNameID: Leptobrachella sungi; scientificName: Leptobrachella sungi; class: Amphibia; order: Anura; family: Megophryidae; genus: Leptobrachella; specificEpithet: sungi; scientificNameAuthorship: (Lathrop, Murphy, Orlov & Ho, 1998); **Location:** country: Vietnam; countryCode: VN; stateProvince: Son La; county: Phu Yen; municipality: Muong Do; locality: near Tan Do Village; verbatimElevation: 980 m; verbatimLatitude: 21°11.89'N; verbatimLongitude: 103°43.42'E; verbatimCoordinateSystem: WGS84; **Event:** eventDate: August 28, 2015; eventRemarks: collected by H.V. Tu and C.K.P.D. Kham; **Record Level:** language: en; collectionCode: Amphibians; basisOfRecord: Preserved Specimen
- **Type status:** Other material. **Occurrence:** catalogNumber: TBU MD.2016.117; individualCount: 1; sex: female; lifeStage: adult; **Taxon:** scientificNameID: Leptobrachella sungi; scientificName: Leptobrachella sungi; class: Amphibia; order: Anura; family: Megophryidae; genus: Leptobrachella; specificEpithet: sungi; scientificNameAuthorship: (Lathrop, Murphy, Orlov & Ho, 1998); **Location:** country: Vietnam; countryCode: VN; stateProvince: Son La; county: Phu Yen; municipality: Muong Do; locality: near Tan Do Village; verbatimElevation: 980 m; verbatimLatitude: 21°11.89'N; verbatimLongitude: 103°43.42'E; verbatimCoordinateSystem: WGS84; **Event:** eventDate: August 28, 2015; eventRemarks: collected by H.V. Tu and C.K.P.D. Kham; **Record Level:** language: en; collectionCode: Amphibians; basisOfRecord: Preserved Specimen
- **Type status:** Other material. **Occurrence:** catalogNumber: TBU MD.2016.125; individualCount: 1; sex: female; lifeStage: adult; **Taxon:** scientificNameID: Leptobrachella sungi; scientificName: Leptobrachella sungi; class: Amphibia; order: Anura; family: Megophryidae; genus: Leptobrachella; specificEpithet: sungi; scientificNameAuthorship: (Lathrop, Murphy, Orlov & Ho, 1998); **Location:** country: Vietnam; countryCode: VN; stateProvince: Son La; county: Phu Yen; municipality: Muong Do; locality: near Tan Do Village; verbatimElevation: 980 m; verbatimLatitude: 21°11.89'N; verbatimLongitude: 103°43.42'E; verbatimCoordinateSystem: WGS84; **Event:** eventDate: August 28, 2015; eventRemarks: collected by H.V. Tu and C.K.P.D. Kham; **Record Level:** language: en; collectionCode: Amphibians; basisOfRecord: Preserved Specimen

#### Description

Morphological characters of the specimens (n = 3 females) from Son La Province agreed with the description of Lathrop et al. (1998): SVL 55.9–56.8 mm; head longer than wide (HL 22.2–23.0 mm, HW 21.4–22.0); snout distinctly pointed in dorsal view, longer than eye diameter (SL 8.6–8.9 mm, ED 6.8–7.0 mm); nostrils oval, on lateral side, closer to the tip of snout than to eye (NS 3.8–4.1 mm, EN 4.5–5.0 mm); canthus rostralis distinct, loreal region concave; interorbital distance wider than upper eyelid width and internarial distance (IOD 6.5–6.8 mm, UEW 4.6–5.0 mm, IN 4.0–4.4 mm); eye large, approximately twice diameter of tympanum (TD 3.2–3.5 mm); tympanum round; vomerine teeth absent; tongue notched posteriorly.

Forelimbs. Forearm robust; relative finger lengths I = II = IV < III, tips of fingers not enlarged into discs; webbing absent; subarticular tubercles indistinct; palmar tubercles round, in contact, inner one very large.

Hindlimbs. Tibia longer than thigh (FL 22.0–23.9 mm, TL 23.0–24.2 mm); relative toe lengths I < II < V < III < IV; webbing rudimentary between toes I–IV and absent between IV and V; subarticular tubercles indistinct; inner metatarsal tubercle oval; outer metatarsal tubercle absent; tibiotarsal articulation reaching to orbit when limb adpressed along body.

Skin. Dorsal surface of head and body, upper part of flanks with tubercles; upper eyelid granular; supratympanic fold distinct, extending from posterior corner of eye to a point behind articulation of jaw; dorsolateral fold absent; ventral surface smooth.

**Figure 3.**
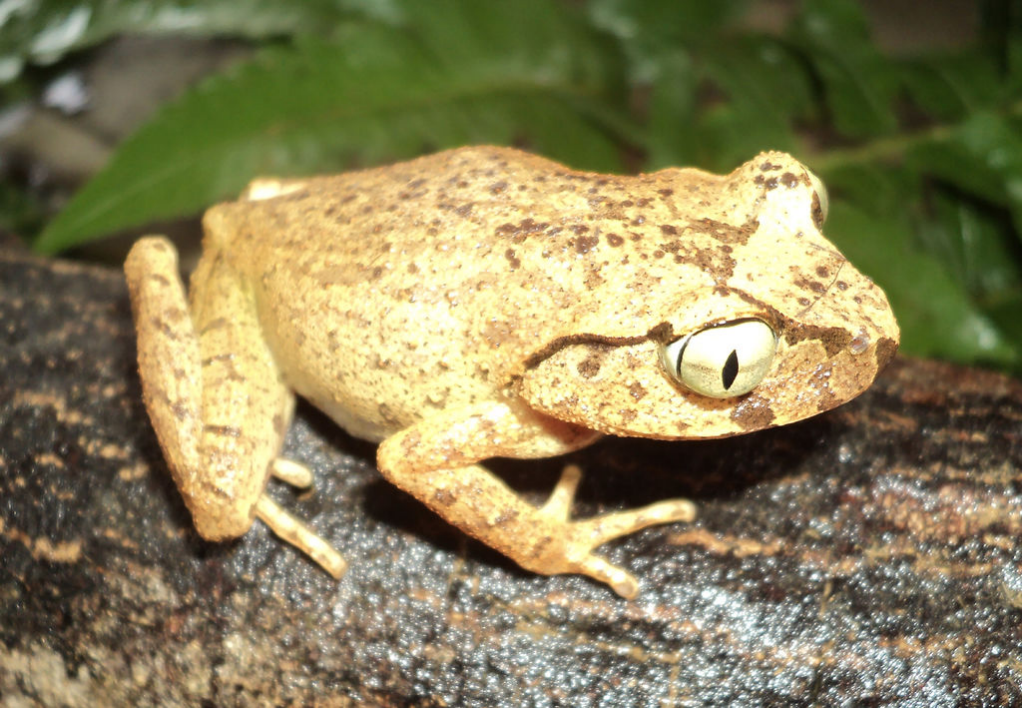
*Leptobrachella sungi* (TBU MD.2015.116, adult female) from Son La Province, Vietnam. Photo by H.V. Tu.

Colouration in life. Dorsal surface of head brown grey with a dark brown marking between eyes; canthus and supratympanic fold dark brown; upper lip with dark brown bars; limbs with dark brown transverse bars; ventral surface orange (Fig. 3).

#### Distribution

In Vietnam, this species has been recorded from Lao Cai, Dien Bien, Yen Bai, and Vinh Phuc provinces (Nguyen et al. 2009). Our new record in Son La Province filled the distribution gap of the species in northwestern Vietnam. Elsewhere, this species is known from China (Frost 2019).

#### Ecology

The specimens were found at night, between 19:00 and 22:00 h, on the side of the streams in the secondary forest.

### Megophrys feae

Boulenger, 1887

85EF46FA-FB49-5863-B36E-3B7159D6174B

#### Materials

- **Type status:** Other material. **Occurrence:** catalogNumber: TBU MD.2015.174; individualCount: 1; sex: male; lifeStage: adult; **Taxon:** scientificNameID: Megophrys feae; scientificName: Megophrys feae; class: Amphibia; order: Anura; family: Megophryidae; genus: Megophrys; specificEpithet: Megophrys; scientificNameAuthorship: Boulenger, 1887; **Location:** country: Vietnam; countryCode: VN; stateProvince: Son La; county: Phu Yen; municipality: Muong Do; locality: near Kieng Village; verbatimElevation: 790 m; verbatimLatitude: 21°11.25'N; verbatimLongitude: 103°45.79'E; verbatimCoordinateSystem: WGS84; **Event:** eventDate: September 4, 2015; eventRemarks: collected by H.V. Tu and C.K.P.D. Kham; **Record Level:** language: en; collectionCode: Amphibians; basisOfRecord: Preserved Specimen

#### Description

Morphological characters of the specimen (n = 1 male) from Son La Province agreed with the descriptions of Boulenger (1887), Inger et al. (1999) and Fei et al. (2009): SVL 96.5 mm; head wider than (HL 37.6 mm, HW 44.8 mm, HL/SVL 39%, HW/SVL 46%); snout round (SL 10.9 mm), longer than horizontal diameter of eye (EL 8.1 mm); canthus rostralis feeble; nostril lateral, slightly closer to eye than to tip of snout (NS 5.8 mm, EN 5.5 mm); interorbital space flat, broader than upper eyelid and internarial distance (IOD 12.0 mm, UEW 5.7 mm, IN 9.5 mm); tympanum indistinct; vomerine teeth present; tongue heart–shaped, slightly notched posteriorly; vocal sac present.

Forelimb short (FLL 47.2 mm); relative finger lengths I < II < IV < III; fingers dermal fringe absent, free of webbing; tips of fingers round, not swollen; subarticular tubercles indistinct; palmar tubercles indistinct; nuptial pads present.

Hindlimb (HLL 127.0 mm); tibia shorter than thigh (TL 41.1 mm, FL 42.3 mm); relative toe lengths I < II < V < III < IV; tips of toes round; toes without dermal fringe; webbing rudimental; inner metatarsal tubercle distinct; subarticular tubercles indistinct; tibiotarsal articulation reaching to commissure of the jaws.

Skin. Dorsal surface smooth with some small warts; upper eyelid with tubercles, one of which being larger; supratympanic fold present, from posterior edge of eye to axilla; ventral surface smooth.

**Figure 4.**
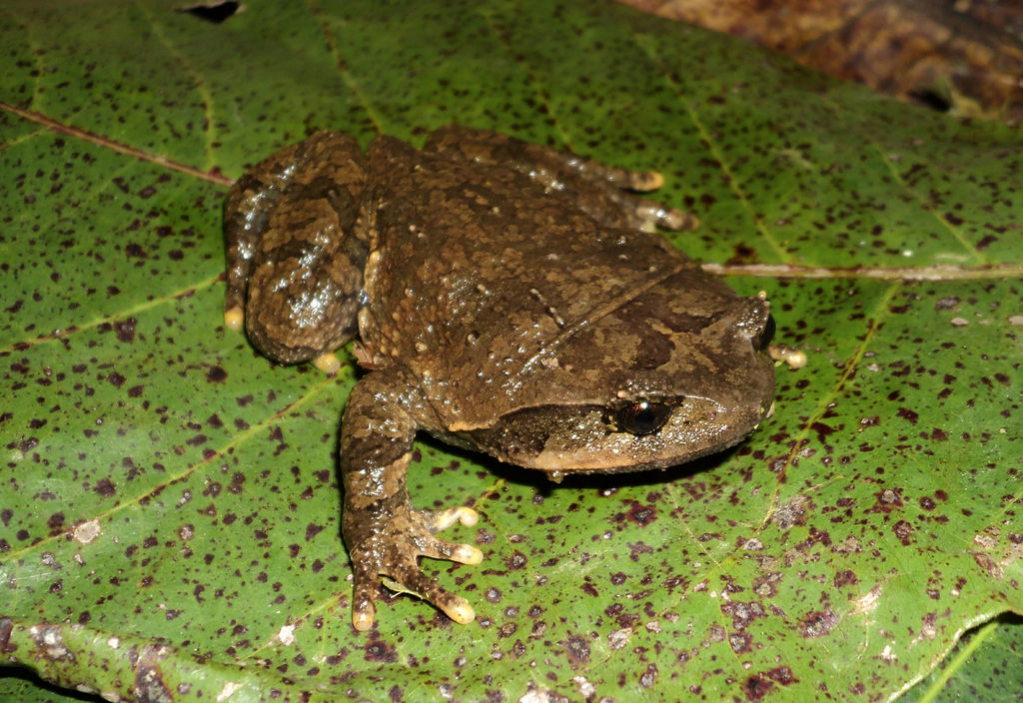
*Megophrys feae* (TBU MD.2015.174, adult male) from Son La Province, Vietnam. Photo by A.V. Pham.

Colouration in life. Dorsal surface brown olive; supratympanic fold edged in dark brown below; upper lip light brown; ventral surface dark brown with some whitish dots (Fig. 4).

#### Distribution

In Vietnam, this species has been recorded from Lao Cai, Yen Bai, Ha Giang, Cao Bang, Lang Son, Vinh Phuc and Bac Giang provinces (Nguyen et al. 2009). Our new record in Son La Province filled the distribution gap of the species in northwestern Vietnam. Elsewhere, this species has been recorded from southern China, Myanmar and Thailand (Frost 2019).

#### Ecology

The specimen was found at 20:15 h on a rock in a stream. The surrounding habitat was mixed secondary forest composed of medium hardwoods and shrubs.

### Megophrys jingdongensis

Fei & Ye, 1983

0149EB24-D39A-570F-83EC-2F2AECA54CE5

#### Materials

- **Type status:** Other material. **Occurrence:** catalogNumber: TBU ML.2017. 9; individualCount: 1; sex: male; lifeStage: adult; **Taxon:** scientificNameID: Megophrys jingdongensis; scientificName: Megophrys jingdongensis; class: Amphibia; order: Anura; family: Megophryidae; genus: Megophrys; specificEpithet: jingdongensis; scientificNameAuthorship: Fei & Ye, 1983; **Location:** country: Vietnam; countryCode: VN; stateProvince: Son La; county: Muong La; municipality: Ngoc Chien; locality: near Nam Nghiep Village; verbatimElevation: 1990 m; verbatimLatitude: 21°19.65'N; verbatimLongitude: 103°36.66'E; verbatimCoordinateSystem: WGS84; **Event:** eventDate: April 16, 2017; eventRemarks: collected by N.B. Song and T.V. Dau; **Record Level:** language: en; collectionCode: Amphibians; basisOfRecord: Preserved Specimen
- **Type status:** Other material. **Occurrence:** catalogNumber: TBU ML.2017. 11; individualCount: 1; sex: female; lifeStage: adult; **Taxon:** scientificNameID: Megophrys jingdongensis; scientificName: Megophrys jingdongensis; class: Amphibia; order: Anura; family: Megophryidae; genus: Megophrys; specificEpithet: jingdongensis; scientificNameAuthorship: Fei & Ye, 1983; **Location:** country: Vietnam; countryCode: VN; stateProvince: Son La; county: Muong La; municipality: Ngoc Chien; locality: near Nam Nghiep Village; verbatimElevation: 1990 m; verbatimLatitude: 21°19.65'N; verbatimLongitude: 103°36.66'E; verbatimCoordinateSystem: WGS84; **Event:** eventDate: April 16, 2017; eventRemarks: collected by N.B. Song and T.V. Dau; **Record Level:** language: en; collectionCode: Amphibians; basisOfRecord: Preserved Specimen
- **Type status:** Other material. **Occurrence:** catalogNumber: TBU XV.2017.118; individualCount: 1; sex: female; lifeStage: adult; **Taxon:** scientificNameID: Megophrys jingdongensis; scientificName: Megophrys jingdongensis; class: Amphibia; order: Anura; family: Megophryidae; genus: Megophrys; specificEpithet: jingdongensis; scientificNameAuthorship: Fei & Ye, 1983; **Location:** country: Vietnam; countryCode: VN; stateProvince: Son La; county: Bac Yen; municipality: Xim Vang; locality: near Nam Nghiep Village; verbatimElevation: 1650 m; verbatimLatitude: 21°23.05'N; verbatimLongitude: 104°23.21'E; verbatimCoordinateSystem: WGS84; **Event:** eventDate: May 1, 2017; eventRemarks: collected by Q.T. Bui and T.V. Dau; **Record Level:** language: en; collectionCode: Amphibians; basisOfRecord: Preserved Specimen
- **Type status:** Other material. **Occurrence:** catalogNumber: IEBR 4558; individualCount: 1; sex: male; lifeStage: adult; **Taxon:** scientificNameID: Megophrys jingdongensis; scientificName: Megophrys jingdongensis; class: Amphibia; order: Anura; family: Megophryidae; genus: Megophrys; specificEpithet: jingdongensis; scientificNameAuthorship: Fei & Ye, 1983; **Location:** country: Vietnam; countryCode: VN; stateProvince: Son La; county: Bac Yen; municipality: Xim Vang; locality: near Nam Nghiep Village; verbatimElevation: 1650 m; verbatimLatitude: 21°23.05'N; verbatimLongitude: 104°23.21'E; verbatimCoordinateSystem: WGS84; **Event:** eventDate: May 1, 2017; eventRemarks: collected by Q.T. Bui and T.V. Dau; **Record Level:** language: en; collectionCode: Amphibians; basisOfRecord: Preserved Specimen

#### Description

Morphological characters of the specimens (n = 4) from Son La Province agreed with the descriptions of Fei et al. (2009) and Nguyen et al. (2016): SVL 49.0–50.0 mm in males (n = 2), 58.5–59.7 mm in females (n = 2); head wider than long (males: HL 17.5–17.7 mm, HW 18.0 mm, HL/SVL 35–36%, HW/SVL 36–37%; females: HL 19.5–20.4 mm, HW 19.9–21.0 mm, HL/SVL 33–35%, HW/SVL 33–36%); snout pointed (SL 6.3–6.5 mm in males, 7.1–7.5 mm in females), longer than horizontal diameter of eye (EL 5.9–6.1 mm in males, 6.9–7.0 mm in females); loreal region concave; nostril lateral, closer to eye than to tip of snout (NS 3.2 mm, EN 2.5–2.6 mm in males; NS 3.6–3.7 mm, EN 2.7–2.9 mm in females); interorbital space flat, slightly broader than upper eyelid and internarial distance (IOD 4.9–5.1 mm, UEW 4.2–5.0 mm, IN 4.8–5.0 mm in males; IOD 6.0 mm, UEW 4.9–5.3 mm, IN 5.7–5.9 mm in females); tympanum distinct (TD 2.8–2.9 mm in males, 3.0–3.1 mm in females) less than half of eye diameter (TD/EL 47–48% in males, 43–45% in females); vomerine teeth present; tongue heart–shaped, round posteriorly; vocal sacs present in males.

Forelimb slender (FLL 29.0–29.3 mm in males, 31.9–34.0 mm in females); relative finger lengths IV ≤ II < I < III; fingers without dermal fringe, free of webbing; tips of fingers round, not swollen; subarticular tubercle indistinct; palmar tubercles indistinct; nuptial pads weak and nuptial spines on first and second fingers.

Hindlimb slender, long (HLL 84.9–87.3 mm in males, 100.3–103.0 mm in females); tibia longer than thigh (TL 27.2–27.7 mm, FL 25.0–25.8 mm in males; TL 30.3–31.6 mm, FL 29.2–29.8 mm in females); relative toe lengths I < II < V < III < IV; tips of toes round, slightly swollen; webbing formula I ¼ – 1II 1/3 – 2III1 ½ – 2IV2 – ½ V; toes with weak dermal fringe; inner metatarsal tubercle distinct; subarticular tubercles indistinct; tibio-tarsal articulation reaching to anterior corner of eye.

Skin. Dorsal surface of head and body smooth; supratympanic fold extending from posterior edge of eye to axilla; upper lips, around tympanum and upper eyelid with small spines; flanks with some tubercles; surrounding of cloaca with tubercles; dorsolateral folds present; and ventral surface smooth.

**Figure 5.**
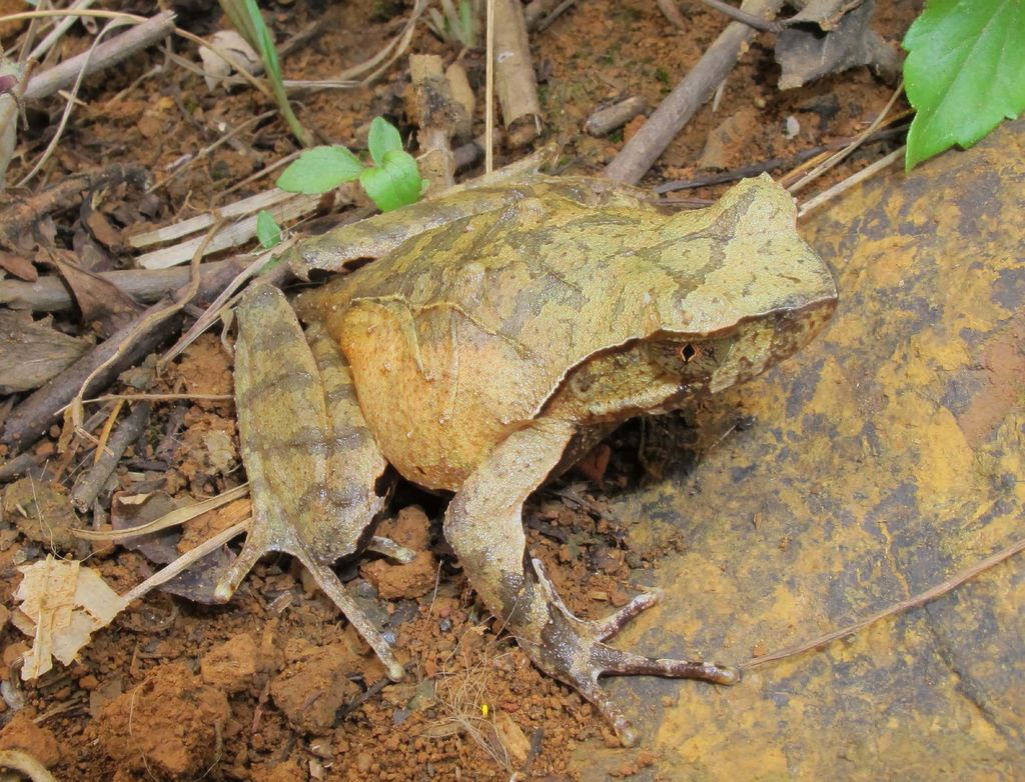
*Megophrys jingdongensis* (TBU ML.2017.9, adult female) from Son La Province, Vietnam. Photo by A.V. Pham.

Colouration in life. Dorsal surface brown to light yellowish-brown; a darker brown triangular marking with a yellow central blotch between eyes; chin, chest and anterior part of belly dark brown in males or light brown with erratic dark brown blotches in females, a round white spot on each side of the chest present; posterior part of belly and ventral surface of thighs cream to dirty white; posterior part of thighs dark brown with some light blotches (Fig. 5).

#### Distribution

In Vietnam, this species has been recorded from Lao Cai and Ha Giang provinces (Nguyen et al. 2009, Nguyen et al. 2016). Our new record in Son La Province is approximately 70 km and 150 km distant from Lao Cai and Ha Giang provinces, respectively. Elsewhere, this species is known from China (Frost 2019).

#### Ecology

The specimens were found on the edge of streams, between 19:00 and 21:30. The surrounding habitat was secondary forest, composed of larger hardwoods and shrubs.

### Megophrys microstoma

(Boulenger, 1903)

A29047B5-4347-5AFE-A636-CF6D7E0EF651

#### Materials

- **Type status:** Other material. **Occurrence:** catalogNumber: TBU SL.2016.419; individualCount: 1; sex: male; lifeStage: adult; **Taxon:** scientificNameID: Megophrys microstoma; scientificName: Megophrys microstoma; class: Amphibia; order: Anura; family: Megophryidae; genus: Megophrys; specificEpithet: microstoma; scientificNameAuthorship: (Boulenger, 1903); **Location:** country: Vietnam; countryCode: VN; stateProvince: Son La; county: Phu Yen; municipality: Muong Bang; locality: near Cai Village; verbatimElevation: 680 m; verbatimLatitude: 21°07.18'N; verbatimLongitude: 103°34.06'E; verbatimCoordinateSystem: WGS84; **Event:** eventDate: October 26, 2017; eventRemarks: collected by A. V. Pham, T. Q. L. Hoang, S. V. Cao, H. V. Cam, and C. V. Cao; **Record Level:** language: en; collectionCode: Amphibians; basisOfRecord: Preserved Specimen
- **Type status:** Other material. **Occurrence:** catalogNumber: TBU SL.2016.483; individualCount: 1; sex: male; lifeStage: adult; **Taxon:** scientificNameID: Megophrys microstoma; scientificName: Megophrys microstoma; class: Amphibia; order: Anura; family: Megophryidae; genus: Megophrys; specificEpithet: microstoma; scientificNameAuthorship: (Boulenger, 1903); **Location:** country: Vietnam; countryCode: VN; stateProvince: Son La; county: Phu Yen; municipality: Gia Phu; locality: near Nhot Village; verbatimElevation: 990 m; verbatimLatitude: 21°12.38'N; verbatimLongitude: 103°34.06'E; verbatimCoordinateSystem: WGS84; **Event:** eventDate: October 29, 2017; eventRemarks: collected by A. V. Pham and H. V. Cam; **Record Level:** language: en; collectionCode: Amphibians; basisOfRecord: Preserved Specimen

#### Description

Morphological characters of specimens (n = 2 males) from Son La Province agreed with the descriptions of Ohler (2003) and Hecht et al. (2013): SVL 37.2–39.0 mm; head small, wider than long (HL 10.4–10.6 mm, HW 10.8–11.0 mm, HL/SVL 27–28%, HW/SVL 28–29%); snout truncate, protruding, projecting beyond upper jaw (SL 3.9–4.1 mm), shorter than horizontal diameter of eye (EL 4.3–4.6 mm); loreal region vertical, concave; nostril lateral, closer to tip of snout than to eye (NS 1.4 mm, EN 2.0–2.3 mm); interorbital space flat, narrower than upper eyelid and internarial distance (IOD 2.4–2.6 mm, UEW 3.5–3.9 mm, IN 2.9–3.2 mm); tympanum round, distinct (TD 2.9–3.0); vomerine teeth absent; tongue round; vocal sacs present in males.

Forelimb slender (FLL 23.5–24.5 mm); relative finger lengths I < II = IV < III, tips of fingers round, not enlarged; fingers free of webbing; subarticular tubercles absent; palmar tubercles indistinct.

Hindlimbs slender (HLL 52.9–57.1 mm); tibia longer than thigh (TL 16.9–18.0 mm, FL 16.6–17.1 mm); relative toe lengths I < II < V < III < IV; tips of toes round, slightly swollen; webbing rudimentary; subarticular tubercles indistinct; metatarsal tubercle indistinct; tibio-tarsal articulation reaching to eye.

Skin. Dorsal surface shagreened, with symmetric glandular ridges; flanks shagreened covered with granules; ventral surface smooth.

**Figure 6.**
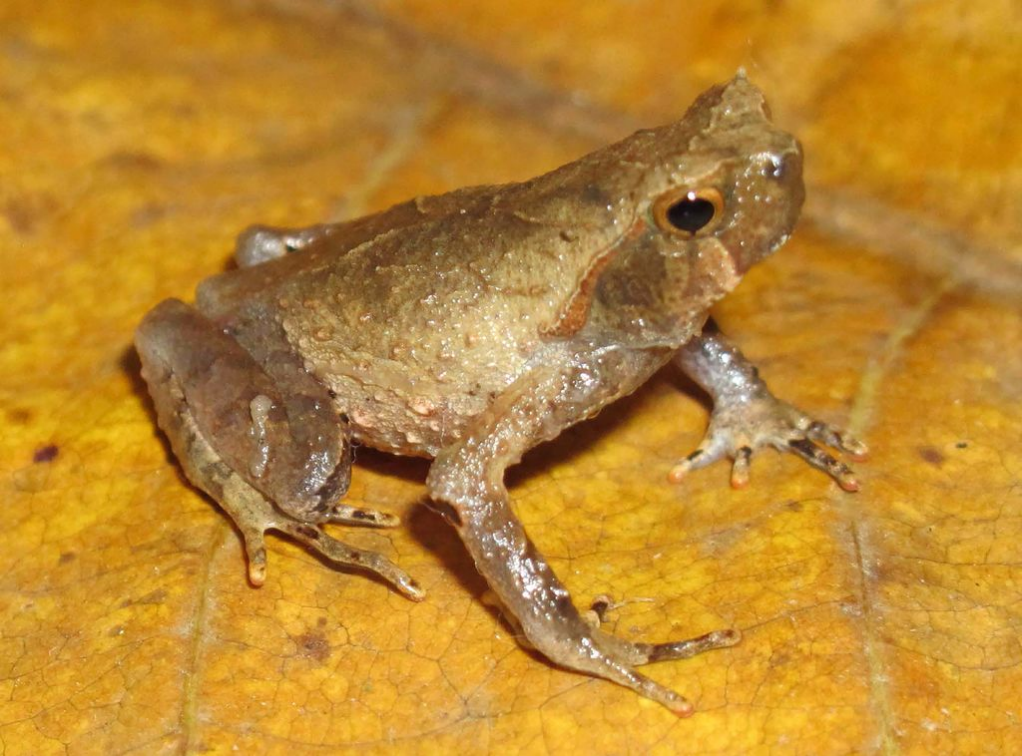
*Megophrys microstoma* (TBU SL.2016.419, adult male) from Son La Province, Vietnam. Photo by A.V. Pham

Colouration in life. Dorsum greyish or light brown, upper surface of limbs with transverse dark brown bars; flanks with some small black spots; ventral surface cream with dark brown marbling (Fig. 6).

#### Distribution

In Vietnam, this species has been recorded from Lao Cai and Ha Giang in the North southwards to Dak Lak and Lam Dong provinces (Nguyen et al. 2009, Hecht et al. 2013). Our new record in Son La Province filled the distribution gap of the species in northwestern Vietnam. Elsewhere, the species is known from China, Laos, Thailand and Cambodia (Frost 2019).

#### Ecology

The specimens were found between 19:30 and 22:00 h on branches or stones near rocky streams. The surrounding habitat was secondary forest composed of medium hardwoods and shrubs.

### Megophrys parva

(Boulenger, 1893)

E1387D99-5005-5EA1-BB48-A91CED368744

#### Materials

- **Type status:** Other material. **Occurrence:** catalogNumber: TBU PAE.705; individualCount: 1; sex: male; lifeStage: adult; **Taxon:** scientificNameID: Megophrys parva; scientificName: Megophrys parva; class: Amphibia; order: Anura; family: Megophryidae; genus: Megophrys; specificEpithet: parva; scientificNameAuthorship: (Boulenger, 1893); **Location:** country: Vietnam; countryCode: VN; stateProvince: Son La; county: Thuan Chau; municipality: Chieng Bom; locality: near Huoi Pu Village; verbatimElevation: 1290 m; verbatimLatitude: 21°23.76'N; verbatimLongitude: 103°38.55'E; verbatimCoordinateSystem: WGS84; **Event:** eventDate: June 19, 2014; eventRemarks: collected by A. V. Pham, H. V. Tu and C. K. P. D. Kham; **Record Level:** language: en; collectionCode: Amphibians; basisOfRecord: Preserved Specimen
- **Type status:** Other material. **Occurrence:** catalogNumber: TBU PAE.711; individualCount: 1; sex: female; lifeStage: adult; **Taxon:** scientificNameID: Megophrys parva; scientificName: Megophrys parva; class: Amphibia; order: Anura; family: Megophryidae; genus: Megophrys; specificEpithet: parva; scientificNameAuthorship: (Boulenger, 1893); **Location:** country: Vietnam; countryCode: VN; stateProvince: Son La; county: Thuan Chau; municipality: Chieng Bom; locality: near Huoi Pu Village; verbatimElevation: 1290 m; verbatimLatitude: 21°23.76'N; verbatimLongitude: 103°38.55'E; verbatimCoordinateSystem: WGS84; **Event:** eventDate: June 19, 2014; eventRemarks: collected by A. V. Pham, H. V. Tu and C. K. P. D. Kham; **Record Level:** language: en; collectionCode: Amphibians; basisOfRecord: Preserved Specimen
- **Type status:** Other material. **Occurrence:** catalogNumber: TBU PAE.712; individualCount: 1; sex: female; lifeStage: adult; **Taxon:** scientificNameID: Megophrys parva; scientificName: Megophrys parva; class: Amphibia; order: Anura; family: Megophryidae; genus: Megophrys; specificEpithet: parva; scientificNameAuthorship: (Boulenger, 1893); **Location:** country: Vietnam; countryCode: VN; stateProvince: Son La; county: Thuan Chau; municipality: Chieng Bom; locality: near Huoi Pu Village; verbatimElevation: 1290 m; verbatimLatitude: 21°23.76'N; verbatimLongitude: 103°38.55'E; verbatimCoordinateSystem: WGS84; **Event:** eventDate: June 19, 2014; eventRemarks: collected by A. V. Pham, H. V. Tu and C. K. P. D. Kham; **Record Level:** language: en; collectionCode: Amphibians; basisOfRecord: Preserved Specimen
- **Type status:** Other material. **Occurrence:** catalogNumber: TBU SC.2015.06; individualCount: 1; sex: male; lifeStage: adult; **Taxon:** scientificNameID: Megophrys parva; scientificName: Megophrys parva; class: Amphibia; order: Anura; family: Megophryidae; genus: Megophrys; specificEpithet: parva; scientificNameAuthorship: (Boulenger, 1893); **Location:** country: Vietnam; countryCode: VN; stateProvince: Son La; county: Song Ma; municipality: Huoi Mot; locality: near Pa Tet Village; verbatimElevation: 1270 m; verbatimLatitude: 21°00.89'N; verbatimLongitude: 103°38.12'E; verbatimCoordinateSystem: WGS84; **Event:** eventDate: April 1, 2015; eventRemarks: collected by A. V. Pham and D. B. Giang; **Record Level:** language: en; collectionCode: Amphibians; basisOfRecord: Preserved Specimen

#### Description

Morphological characters of the specimens (n = 4) from Son La Province agreed with the descriptions of Boulenger (1893), Bourret (1942), and Fei et al. (2009): SVL 44.5–46.7 mm in males (n = 2), 55.4–56.7 mm in females (n = 2); head longer than wide (males: HL 15.4–16.5 mm, HW 14.8–16.2 mm, HL/SVL 35%, HW/SVL 33–35%; females: HL 18.7 mm, HW 18.0–18.5 mm, HL/SVL 33–34%, HW/SVL 32–33%); snout obliquely truncate, projecting beyond upper jaw (SL 5.9–6.7 mm in males, 6.7–7.0 mm in females), longer than horizontal diameter of eye (EL 5.3–5.9 mm in males, 5.8 mm in females); loreal region concave; nostril lateral, slightly closer to eye than to tip of snout (NS 2.8–3.2 mm, EN 2.7–2.9 mm in males; NS 3.2–3.3 mm, EN 3.1–3.2 mm in females); interorbital space flat, broader than upper eyelid and narrower than internarial distance (IOD 4.6–4.7 mm, UEW 4.1–4.2 mm, IN 5.0–5.5 mm in males; IOD 5.0–5.2 mm, UEW 4.2–4.7 mm, IN 5.5–5.6 mm in females); tympanum distinct (TD 3.5–3.7 mm in males, 4.5 mm in females); vomerine teeth present; tongue heart–shaped, round posteriorly; vocal sacs present in males.

Forelimb slender (FLL 27.0–28.9 mm in males, 31.0–34.1 mm in females); relative finger lengths I ≤ II < IV < III; fingers dermal fringe absent, free of webbing; tips of fingers round, not swollen; subarticular tubercles indistinct; palmar tubercles indistinct; nuptial pads absent in males.

Hindlimb slender, long (HLL 69.1–75.6 mm in males, 85.0–94.0 mm in females); tibia longer than thigh (TL 22.2–23.7 mm, FL 20.5–20.6 mm in males; TL 27.0–29.8 mm, FL 24.4–24.6 mm in females); relative toe lengths I < II < V < III < IV; tips of toes round, slightly swollen; webbing rudimentary; toes without dermal fringe; metatarsal tubercle indistinct; subarticular tubercles indistinct; tibiotarsal articulation reaching to anterior margin of eye.

Skin. Dorsal surface smooth; flanks with small glandular warts; supratympanic fold present, from posterior edge of eye to axilla; around cloaca with small tubercles; ventral surface smooth.

**Figure 7.**
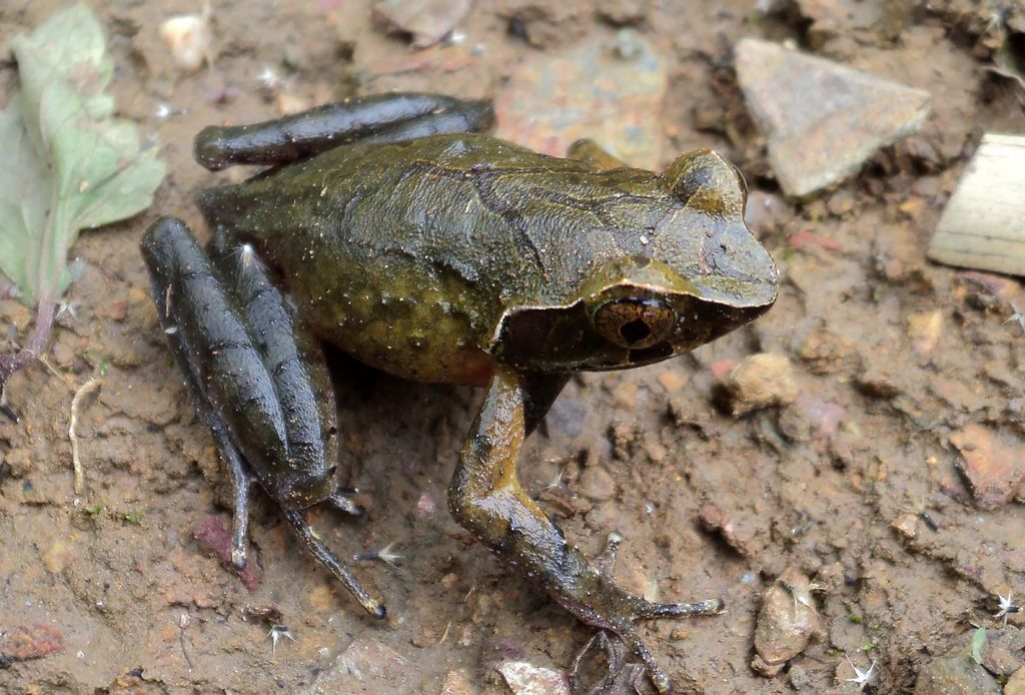
*Megophrys parva* (TBU PAE.705, adult male) from Son La Province, Vietnam. Photo by A.V. Pham.

Colouration in life. Dorsal surface light yellowish-brown; a darker brown triangular marking between eyes; upper lips with vertical dark brown bars; ventral surface whitish, a round white spot on each side of the chest present; ventral surface of limbs reddish (Fig. 7).

#### Distribution

In Vietnam, this species has been recorded from Lao Cai, Lai Chau, Dien Bien, and Ha Giang provinces (Nguyen et al. 2009, Orlov et al. 2015, Luong et al. 2019). Our record in Son La Province is the southernmost record of this species in Vietnam. Elsewhere, this species is known from Nepal, India, Bangladesh, Myanmar, Thailand, China, and Laos (Nguyen et al. 2009, Frost 2019).

#### Ecology

The specimens were found on the edge of streams, between 19:00 and 22:00 h. The surrounding habitat was mixed secondary forest of medium hardwoods and shrubs.

## Discussion

The herpetofauna of Son La Province is imperfectly studied, particularly in remote forest areas. Recent studies on the herpetofauna of Son La Province have revealed a series of new discoveries of amphibians: two new species (*Tylototriton anguliceps* Le, Nguyen, Nishikawa, Nguyen, Pham, Matsui, Bernardes & Nguyen and *Amolops ottorum* Pham, Sung, Pham, Le, Ziegler & Nguyen) and several new country records, for instance, *Leptobrachium masatakasatoi* Matsui, *Leptobrachella eos* (Ohler, Wollenberg, Grosjean, Hendrix, Vences, Ziegler & Dubois), *L. minima* (Taylor), *Megophrys daweimontis* Rao & Yang, *Amolops vitreus* (Bain, Stuart & Orlov), *Nidirana lini* (Chou), and *Sylvirana cubitalis* (Smith) (Le et al. 2014, Pham et al. 2014a, Pham et al. 2014b, Le et al. 2015a, Le et al. 2015b, Pham et al. 2016, Pham et al. 2019). Amongst six newly recorded species from Son La Province, three species were found in the protected areas (i.e. Copia, Sop Cop and Muong La nature reserves) and three other species were recorded in Phu Yen District. Our new findings bring the total species number of the family Megophryidae to 13 in Son La Province (after Nguyen et al. 2009, Pham et al. 2014b, Pham et al. 2016). Further field surveys are likely to reveal a much greater diversity of amphibians from Son La Province, particularly in poorly explored forests of Muong La, Ta Xua, and Xuan Nha nature reserves.

## Supplementary Material

XML Treatment for Leptobrachium ailaonicum

XML Treatment for Leptobrachella sungi

XML Treatment for Megophrys feae

XML Treatment for Megophrys jingdongensis

XML Treatment for Megophrys microstoma

XML Treatment for Megophrys parva
